# Assessment of Confounding Factors Affecting the Tumor Markers SMRP, CA125, and CYFRA21-1 in Serum

**DOI:** 10.4137/bmi.s3927

**Published:** 2010-01-28

**Authors:** Daniel Gilbert Weber, Georg Johnen, Dirk Taeger, Anne Weber, Isabelle Mercedes Gross, Beate Pesch, Thomas Kraus, Thomas Brüning, Monika Gube

**Affiliations:** 1 BGFA—Research Institute of Occupational Medicine, German Social Accident Insurance, Ruhr-University Bochum, Bochum, Germany; 2 Institute for Occupational and Social Medicine, RWTH Aachen University, Aachen, Germany

**Keywords:** SMRP, mesothelin, CA125, CYFRA21-1, mesothelioma, lung cancer, screening

## Abstract

The purpose of this analysis was to evaluate if serum levels of potential tumor markers for the diagnosis of malignant mesothelioma and lung cancer are affected by confounding factors in a surveillance cohort of workers formerly exposed to asbestos. SMRP, CA125, and CYFRA21-1 concentrations were determined in about 1,700 serum samples from 627 workers formerly exposed to asbestos. The impact of factors that could modify the concentrations of the tumor markers was examined with linear mixed models. SMRP values increased with age 1.02-fold (95% CI 1.01–1.03) and serum creatinine concentration 1.32-fold (95% CI 1.20–1.45). Levels differed by study centers and were higher after 40 years of asbestos exposure. CA125 levels increased with longer storage of the samples. CYFRA21-1 values correlated with age 1.02-fold (95% CI 1.01–1.02), serum creatinine 1.21-fold (95% CI 1.14–1.30) and varied by study centers due to differences in sample handling. Tumor marker concentrations are influenced by subject-related factors, sample handling, and storage. These factors need to be taken into account in screening routine.

## Introduction

Malignant mesothelioma (MM) is an aggressive cancer of the serous membranes highly associated with asbestos exposure. The annual number of new MM cases in Western Europe is expected to rise, with a maximum in the next two decades. MM may account for as many as 250,000 deaths in the next 35 years.^1^ Similar trends have been reported in many countries worldwide.^2^ The latency period of the tumor is up to 40 years and symptoms occur in late stages of the disease. Up to date, MM is an incurable disease, but a combination of pemetrexed and cisplatin showed a significantly improved response rate and overall survival.^3^ An earlier diagnosis of tumors generally leads to more effective therapies and the same might be true for MM. Tumor markers may facilitate an early diagnosis of cancer. Measurement of tumor markers in blood is a feasible minimally invasive method and may be suitable for application in screening routine.^4^

A promising tumor marker for MM is soluble mesothelin-related peptides (SMRP).^5^ SMRP has increasingly been used as a marker for MM^6^ and has been found to be elevated in asbestos-exposed patients up to five years before onset of clinical symptoms, suggesting a potential role as a marker for the early diagnosis of MM.^7^ Additionally, SMRP may be helpful in diagnosis of lung cancer (LC).^8^ While a single tumor marker often lacks sufficient sensitivity and specificity, combinations of markers may improve the diagnostic performance. CA125 and CYFRA21-1 are additional candidates for a marker panel based on results in pleural effusions.^9^,^10^

To improve the use of tumor markers as screening tools for the early detection of cancer, their distribution in screening populations with regard to factors that might influence the marker levels has to be considered. Raised SMRP levels are observed in patients with renal failure and hypertension.^5^ CA125 values can increase in patients with cardiac dysfunction.^11^ Tumor markers could also be influenced by age and gender, or factors like sample handling and storage conditions.^12^ Recently, we have shown that SMRP exhibits excellent stability regarding frozen storage^13^ but for CA125 and CYFRA21-1 less details are known. To determine confounding factors with significant influences on marker levels, tumor markers need to be analyzed in pre-clinical samples obtained prior to diagnosis.^4^ As asbestos is the main etiologic factor for MM, surveillance cohorts of asbestos workers represent an appropriate study population for the evaluation of potential confounders.

The objective of this study was to analyze the distribution of serum SMRP, CA125, and CYFRA21-1 in a cohort of workers formerly exposed to asbestos without MM or LC at the beginning of the surveillance and to identify and evaluate potential factors with significant influence on tumor marker levels.

## Material and Methods

### Study population

The study group of 627 German asbestos workers originates from a study of Marczynski et al.^14^ A questionnaire was applied at baseline in 1993. Blood samples were taken annually (1993–1997) during medical examinations. After clotting, serum samples were separated and frozen immediately. In 2005, about 1,700 serum samples were available. A mortality follow-up was conducted until April 2007. One person with diagnosed LC in 1993 was excluded from analysis. Samples from 33 participants obtained after diagnosis of MM or LC were also excluded. All participants provided informed consent. The study was designed according to rules guarding patient privacy and an updated permission from the local ethics committee was obtained in 2005.

### Measurement of SMRP, CA125, and CYFRA21-1

Of 626 participants, at least one serum sample was available for the evaluation of SMRP, CA125, and CYFRA21-1. Serum SMRP was analyzed using the commercial ELISA kit MESOMARK (provided by Fujirebio Diagnostics, Inc., Malvern, PA, USA)^5^ according to Weber et al.^13^ CA125 was analyzed using the ADVIA Centaur System (Bayer Health Care, Wuppertal, Germany) and CYFRA21-1 was analyzed using the Elecsys 2010 System (Roche Diagnostics, Mannheim, Germany) according to the manufacturer’s instructions.

### Measurement of creatinine and CRP

Of 620 participants serum samples were available for the determination of creatinine and C-reactive protein (CRP) using the UniCel DxC 800 (Beckman Coulter, Krefeld, Germany) according to the manufacturer’s instructions. Serum creatinine is an indicator for renal diseases and CRP for hypertension and heart failure.

## Statistical Methods

Linear mixed models were applied to the data to estimate the effects of subject-related and sample-related factors on the marker levels. These models account for the correlated data structure because samples were collected repeatedly from the same subjects over time. Factors selected for analysis comprised age at entry in the surveillance cohort (with 1993 as reference year), gender, smoking status (current, former, and never smoker), duration of asbestos exposure (in decades), alterations of pleura, alterations of lung, creatinine, CRP, study center, storage time (9, 10, 11, 12 years after collection), and later diagnosed MM or LC. Due to the skewed distribution of the marker concentrations these variables were log-transformed. Therefore, the effect estimates and their confidence intervals were re-transformed via the exponential function presenting the factor of change in the marker concentrations.

## Results

At baseline, the median age of the study population (n = 626) was 63 years (range 53–70 years). The majority (91.9%) of the participants were male (n = 575). Only few participants were current smokers (n = 90, 14.4%), whereas the majority were former (n = 348, 55.6%) or never smokers (n = 188, 30.0%). Median duration of asbestos exposure was 19 years (range 1–52 years). HRCT screens revealed that the majority (n = 410, 65.5%) of participants had pleural (plaques and thickenings) and/or lung (fibrotic) alterations.

Table 1 depicts the distribution of marker concentrations by median and inter-quartile range (IQR) and is further stratified by potential factors selected for analysis. Median SMRP concentration in the study group was 0.72 nmol/l (IQR 0.50–1.03 nmol/l). Median CA125 level was 10.40 KIU/l (IQR 7.40–14.95 KIU/l), and median CYFRA21-1 value was 1.09 ng/ml (IQR 0.82–1.41 ng/ml).

The results of the linear mixed model for SMRP, CA125, and CYFRA21-1 are presented in Table 2. The factors gender, smoking status, CRP levels, alterations of pleura or lung, and development of MM had no significant influence on any of the analyzed markers. SMRP concentrations increased 1.02-fold (95% CI 1.01–1.03, p = 0.0014) per age in years and 1.32-fold (95% CI 1.20–1.45, p < 0.0001) per ng/ml serum creatinine. In subjects >65 years median SMRP values (0.82 nmol/l) were significantly higher than in participants ≤65 years (0.67 nmol/l, p = 0.0002). Also, median SMRP values in participants with >40 years of asbestos exposure were elevated (0.98 nmol/l) compared to participants with ≤40 years of asbestos exposure (0.70 nmol/l, p = 0.0002). Further, two centers were present with elevated SMRP levels. SMRP and CYFRA21-1 are not affected by storage time, as the median values show (Table 1). Also, individual time courses did not show any trend (data not shown). However, storage time varied between nine and twelve years and showed a strong impact on the CA125 levels. Compared to the levels after nine years (median 8.90 KIU/l), the CA125 concentration was 1.23-fold (95% CI 1.18–1.29, p < 0.0001) higher after twelve years of storage. CYFRA21-1 correlated with LC (1.42-fold, 95% CI 1.12–1.79, p = 0.0033). Higher CYFRA21-1 values were observed for age (1.02-fold per year, 95% CI 1.01–1.02, p = 0.0005) and rising creatinine values (1.21-fold per ng/ml, 95% CI 1.14–1.30, p < 0.0001). CYFRA21-1 concentrations varied also in study centers.

## Discussion

A major aim in cancer surveillance and screening programs is the diagnosis of the disease at early stages. The use of tumor markers for the early detection of cancer requires knowledge of factors that might influence the marker levels because the behavior of markers in healthy, unaffected subjects largely defines screening thresholds and hence specificity.^4^

We analyzed the influence of potential confounders on serum concentrations of SMRP, CA125, and CYFRA21-1 in a healthy surveillance cohort. The assessment of specificity and sensitivity to detect MM and LC was not focus of this analysis. A detailed discussion about the correlation of SMRP, CA125, and CYFRA21-1 with MM and LC will be presented elsewhere.

The median SMRP concentration of 0.72 nmol/l found among 626 subjects is in accordance with SMRP levels in recent studies with published values of 0.67 nmol/l (n = 86), 0.61 nmol/l (n = 112), and 0.77 nmol/l (n = 26).^15^–^17^ Also, median CA125 (10.4 KIU/l) and CYFRA21-1 (1.09 ng/ml) are in line with levels reported in the literature, e.g. 9.2 KIU/l (n = 86) and 1.00 ng/ml (n = 27), respectively.^15^,^18^ Median marker levels calculated in this study were based on an adequate number of subjects and might be suitable reference values for use in screening programs.

Our analysis shows that the tumor markers are significantly influenced by various factors. A significantly higher level of SMRP was observed in subjects with an asbestos exposure of >40 years. While Pass et al observed no correlation between exposure duration and SMRP levels,^19^ Rodríguez Portal et al suggested recently that increased release of SMRP in serum occurs as a consequence of asbestos exposure.^20^ Here we show that SMRP might indeed be associated with asbestos exposure at least for long-term exposure. As this could be also reflecting an age-related effect, we analyzed the SMRP values in association with both age and duration of asbestos exposure. Higher SMRP values for participants with >40 years of asbestos exposure could be confirmed independently of age (data not shown). However, only 5% (n = 32) of the participants had an asbestos exposure of >40 years. Further analyses need to be done to validate the feasibility of SMRP as a marker of asbestos exposure.

Age was shown to be a statistically significant predictor of SMRP and CYFRA21-1 values. This is in contrast to our previous observation^13^ and the results of Pass et al.^19^ A possible explanation could be the age distribution in the study groups because this surveillance cohort comprised subjects of a median age of 63 years, whereas the previous groups were on average 20 years younger. Our results support a correlation between age and SMRP values, but only at older age. This should be taken into account in surveillance because MM is mainly diagnosed at 61–65 years.^21^

Creatinine is an indicator for renal diseases, which are potential confounders of SMRP.^5^,^22^ A decreased glomerular filtration rate (GFR) correlated with increased SMRP values in a number of MM patients^22^ and with raised CYFRA21-1 levels in patients without evidence of neoplasia.^23^ Here we show that raised SMRP and CYFRA21-1 levels are indeed correlated with increased creatinine levels supporting the observation that renal failure could lead to higher marker levels. Therefore, measurement of creatinine and estimation of GFR should be applied in surveillance to reduce the number of false-positive cases.

In some study centers SMRP and CYFRA21-1 showed altered values whereas CA125 was not affected. This might be caused by different sample handling procedures between blood drawing and freezing of serum. As results can be misleading if conditions of the procedure are not standardized,^24^ the complete procedure, including clotting time and centrifugation, should be performed according to stringent protocols.

Recently, Roe et al raised the question if serum samples analyzed retrospectively are practical for the analysis of the selected markers.^25^ Our results demonstrate that frozen serum samples are indeed suitable for the retrospective analysis of SMRP and CYFRA-21, but not for CA125. For CA125, higher levels were observed after a longer duration of storage (Table 2). This is in agreement with former observations that attribute the changes to storage rather than physiological effects.^26^ Previously, we have indicated that SMRP is stable during long-term storage^13^ and this study confirms our observation. To our knowledge, for CYFRA21-1 this is the first report of stability during long-term storage.

In the first prospective study analyzing SMRP a relatively high false-positive rate was observed by Park et al.^6^ The results indicated that SMRP appears not to be suited as a screening marker for early detection of MM. It would be interesting if the higher number of false-positive cases may be due to factors identified in our study.

This analysis identified factors influencing SMRP, CA125, and CYFRA21-1 concentrations in serum. Age, serum creatinine, duration of asbestos exposure, sample handling procedures and storage time were significant confounders of serum marker levels. Taken these results into account might reduce the number of false-positive cases in screening programs and hence raise specificity.

## Figures and Tables

**Table 1:** Distribution of marker concentrations by median and inter-quartile range (IQR) stratified by potential factors selected for analysis.

		**N**	**SMRP (nmol/l)**		**CA125 (KIU/l)**		**CYFRA21-1 (ng/ml)**	
			**Median**	**IQR**	**Median**	**IQR**	**Median**	**IQR**
Total		626	0.72	0.50–1.03	10.40	7.40–14.95	1.09	0.82–1.41
Age	≤65 years	459	0.67	0.48–0.96	10.50	7.40–15.00	1.06	0.81–1.36
	>65 years	167	0.82	0.57–1.17	10.20	7.70–14.90	1.22	0.89–1.53
Gender	Male	575	0.73	0.51–1.04	10.60	7.40–15.05	1.10	0.82–1.42
	Female	51	0.61	0.43–0.94	9.40	7.50–12.40	1.00	0.87–1.30
Smoking	Never	188	0.66	0.48–0.98	9.85	7.65–14.25	1.12	0.87–1.41
	Former	348	0.74	0.51–1.02	10.88	7.78–15.18	1.07	0.81–1.43
	Current	90	0.79	0.55–1.10	10.00	6.65–14.55	1.08	0.78–1.34
Creatinine	≤1.2 ng/ml	595	0.72	0.50–1.01	10.30	7.40–14.80	1.08	0.82–1.40
	>1.2 ng/ml	25	0.88	0.58–1.93	13.00	7.30–15.40	1.38	0.88–3.37
CRP	≤1.5 ng/ml	600	0.72	0.50–1.03	10.30	7.40–14.83	1.08	0.82–1.41
	>1.5 ng/ml	20	0.81	0.53–1.04	11.73	8.85–20.75	1.22	0.91–2.13
Asbestos exposure	<10 years	31	0.64	0.45–0.95	10.10	7.10–13.40	1.03	0.77–1.31
	11–20 years	137	0.72	0.51–1.00	10.75	8.20–15.90	1.06	0.85–1.43
	21–30 years	121	0.71	0.48–0.95	9.95	6.90–15.40	1.17	0.84–1.45
	31–40 years	138	0.76	0.57–1.11	11.10	8.00–16.40	1.13	0.86–1.48
	>40 years	199	0.98	0.75–1.47	8.90	7.00–14.20	1.29	0.93–1.59
Pleural alterations^1^	Yes	397	0.74	0.51–1.04	10.40	7.50–15.00	1.11	0.86–1.46
	No	229	0.70	0.50–0.99	10.40	7.30–14.50	1.06	0.78–1.37
Lung alterations^1^	Yes	199	0.74	0.53–1.04	10.30	7.30–14.85	1.10	0.86–1.40
	No	427	0.71	0.49–1.03	10.50	7.50–14.95	1.08	0.79–1.42
Study center	1	14	0.78	0.67–1.09	13.05	8.80–18.00	1.36	1.22–2.00
	2	112	0.81	0.56–1.14	11.65	7.75–14.98	1.04	0.79–1.38
	3	148	0.65	0.44–1.00	9.58	6.70–14.10	0.95	0.75–1.27
	4	100	0.74	0.52–0.95	9.60	6.85–15.28	1.22	0.99–1.59
	5	143	0.79	0.51–1.10	10.80	7.80–15.40	1.08	0.82–1.43
	6	109	0.68	0.46–0.94	10.90	7.80–14.90	1.12	0.81–1.46
Time period	9 years^2^	531, 530, 529	0.70	0.50–0.89	8.90	6.35–13.05	1.22	0.95–1.59
	10 years^2^	482, 479, 479	0.70	0.50–1.01	9.50	6.80–14.10	1.17	0.88–1.53
	11 years^2^	440, 438, 437	0.71	0.46–1.02	10.30	7.40–14.20	1.04	0.76–1.37
	12 years	80	0.71	0.48–1.02	11.30	7.70–16.60	1.06	0.78–1.38
Lung cancer	Yes	12	0.90	0.62–1.43	11.80	6.53–24.60	1.57	1.11–2.87
	No	614	0.72	0.50–1.03	10.40	7.50–14.90	1.08	0.82–1.41
Malignant	Yes	20	0.84	0.58–0.99	10.80	7.00–15.58	1.12	0.68–1.40
mesothelioma	No	606	0.72	0.50–1.04	10.40	7.40–14.90	1.09	0.82–1.41

1 Benign alterations of the pleura (plaques and thickenings) and lung (fibrotic) were detected by HRCT.

2 Available number of samples was different for SMRP, CA125, and CYFRA21-1.

**Table 2:** Analysis of potential factors influencing SMRP, CA125, and CYFRA21-1 levels in serum. Values of exp(β) >1.00 indicate a positive association between analyzed factor and marker, values <1.00 a negative association. Significant changes are marked in bold.

		**SMRP (nmol/l)**			**CA125 (KIU/l)**			**CYFRA21-1 (ng/ml)**		
		**exp(β)**	**95% CI**	**P-value**	**exp(β)**	**95% CI**	**P-value**	**exp(β)**	**95% CI**	**P-value**
Intercept		0.13	0.06–0.29	<0.0001	6.39	2.73–14.96	<0.0001	0.37	0.21–0.65	0.0005
Age (years)		**1.02**	**1.01–1.03**	**0.0014**	1.00	0.99–1.02	0.5824	**1.02**	**1.01–1.02**	**0.0005**
Gender	Male	1.03	0.86–1.22	0.7565	1.05	0.87–1.27	0.6341	0.94	0.83–1.06	0.3061
	Female	1.00	–	–	1.00	–	–	1.00	–	–
Smoking	Never	1.00	–	–	1.00	–	–	1.00	–	–
	Former	0.97	0.88–1.08	0.6206	1.05	0.94–1.18	0.3834	0.95	0.88–1.03	0.1985
	Current	1.12	0.97–1.29	0.1338	0.94	0.80–1.10	0.4424	0.92	0.83–1.02	0.1185
Creatinine (ng/ml)		**1.32**	**1.20–1.45**	<**0.0001**	0.97	0.88–1.08	0.6159	**1.21**	**1.14–1.30**	<**0.0001**
CRP (ng/ml)		0.99	0.94–1.06	0.8624	1.03	0.97–1.10	0.3000	1.02	0.97–1.06	0.4496
Asbestos exposure	<10 years	1,00	–	–	1.00	–	–	1.00	–	–
	11–20 years	1.04	0.92–1.18	0.4850	**1.15**	**1.01–1.32**	**0.0397**	1.05	0.97–1.15	0.2408
	21–30 years	0.92	0.80–1.05	0.2238	1.05	0.90–1.22	0.5188	1.03	0.93–1.13	0.5699
	31–40 years	1.05	0.91–1.21	0.5144	1.08	0.92–1.26	0.3402	1.01	0.92–1.12	0.7990
	>40 years	**1.29**	**1.03–1.61**	**0.0265**	0.92	0.72–1.18	0.5013	1.01	0.86–1.19	0.8657
Pleural alterations^1^		0.98	0.89–1.08	0.6470	1.09	0.98–1.22	0.1161	1.07	0.99–1.14	0.0794
Lung alterations^1^		1.04	0.94–1.15	0.4566	1.00	0.89–1.12	0.9841	0.99	0.92–1.06	0.6933
Study center	1	1.13	0.82–1.56	0.4518	1.17	0.82–1.67	0.3967	**1.27**	**1.00–1.62**	**0.0460**
	2	**1.16**	**1.00–1.35**	**0.0482**	1.00	0.85–1.17	0.9695	0.96	0.86–1.07	0.4845
	3	1.03	0.90–1.18	0.7008	0.92	0.80–1.07	0.2994	**0.87**	**0.78–0.96**	**0.0042**
	4	1.06	0.91–1.23	0.4456	0.99	0.84–1.17	0.9047	**1.13**	**1.01–1.25**	**0.0295**
	5	**1.20**	**1.05–1.38**	**0.0095**	1.02	0.88–1.19	0.7841	1.01	0.91–1.11	0.8807
	6	1.00	–	–	1.00	–	–	1.00	–	–
Storage time	9 years	1.00	–	–	1.00	–	–	1.00	–	–
	10 years	1.01	0.95–1.07	0.7684	**1.07**	**1.03–1.12**	**0.0003**	1.04	0.97–1.11	0.2669
	11 years	1.00	0.95–1.07	0.8972	**1.17**	**1.11–1.22**	<**0.0001**	0.94	0.88–1.01	0.0983
	12 years	1.01	0.95–1.08	0.6634	**1.23**	**1.18–1.29**	<**0.0001**	0.95	0.89–1.02	0.1738
Lung cancer		1.24	0.90–1.71	0.1871	1.06	0.75–1.51	0.7268	**1.42**	**1.12–1.79**	**0.0033**
Malignant mesothelioma		1.06	0.83–1.34	0.6599	0.95	0.73–1.24	0.6959	0.94	0.79–1.12	0.4766

1 Benign alterations of the pleura (plaques and thickenings) and lung (fibrotic) were detected by HRCT.

## References

[1] Peto J Decarli A La Vecchia C Levi F Negri E The European mesothelioma epidemic Br J Cancer 1999 79 3 4 666 672 10.1038/sj.bjc.6690105 PMC2362439 10027347

[2] Scherpereel A Lee YG Biomarkers for mesothelioma Curr Opin Pulm Med 2007 13 4 339 443 17534183 10.1097/MCP.0b013e32812144bb

[3] Vogelzang NJ Rusthoven JJ Symanowski J Phase III study of pemetrexed in combination with cisplatin versus cisplatin alone in patients with malignant pleural mesothelioma J Clin Oncol 2003 21 14 2636 44 12860938 10.1200/JCO.2003.11.136

[4] Lowe KA Shah C Wallace E Effects of personal characteristics on serum CA125, mesothelin, and HE4 levels in healthy postmenopausal women at high-risk for ovarian cancer Cancer Epidemiol Biomarkers Prev 2008 17 9 2480 7 18768519 10.1158/1055-9965.EPI-08-0150 PMC2632599

[5] Beyer HL Geschwindt RD Glover CL MESOMARK: a potential test for malignant pleural mesothelioma Clin Chem 2007 53 4 666 72 17289801 10.1373/clinchem.2006.079327

[6] Park EK Sandrini A Yates DH Soluble mesothelin-related protein in an asbestos-exposed population: the dust diseases board cohort study Am J Respir Crit Care Med 2008 178 8 832 7 18583574 10.1164/rccm.200802-258OC

[7] Robinson BW Creaney J Lake R Mesothelin-family proteins and diagnosis of mesothelioma Lancet 2003 362 9396 1612 6 14630441 10.1016/S0140-6736(03)14794-0

[8] Cristaudo A Foddis R Vivaldi A Clinical significance of serum mesothelin in patients with mesothelioma and lung cancer Clin Cancer Res 2007 13 17 5076 81 17785560 10.1158/1078-0432.CCR-07-0629

[9] Paganuzzi M Onetto M Marroni P Diagnostic value of CYFRA21–1 tumor marker and CEA in pleural effusion due to mesothelioma Chest 2001 119 4 1138 42 11296181 10.1378/chest.119.4.1138

[10] Porcel JM Vives M Esquerda A Salud A Perez B Rodriguez-Panadero F Use of a panel of tumor markers (carcinoembryonic antigen, cancer antigen 125, carbohydrate antigen 15–3 and cytokeratin 19 fragments) in pleural fluid for the differential diagnosis of benign and malignant effusions Chest 2004 126 6 1757 63 15596670 10.1378/chest.126.6.1757

[11] Nagele H Bahlo M Klapdor R Schaeperkoetter D Rodiger W CA125 and its relation to cardiac function Am Heart J 1999 137 6 1044 9 10347329 10.1016/s0002-8703(99)70360-1

[12] Fraser CG Inherent biological variation and reference values Clin Chem Lab Med 2004 42 7 758 64 15327011 10.1515/CCLM.2004.128

[13] Weber DG Taeger D Pesch B Kraus T Bruning T Johnen G Soluble mesothelin-related peptides (SMRP)—High stability of a potential tumor marker for mesothelioma Cancer Biomark 2007 3 6 287 92 18048966 10.3233/cbm-2007-3602

[14] Marczynski B Rozynek P Kraus T Schlosser S Raithel HJ Baur X Levels of 8-hydroxy-2’-deoxyguanosine in DNA of white blood cells from workers highly exposed to asbestos in Germany Mutat Res 2000 468 2 195 202 10882896 10.1016/s1383-5718(00)00053-x

[15] Creaney J van Bruggen I Hof M Combined CA125 and Mesothelin Levels for the Diagnosis of Malignant Mesothelioma Chest 2007 132 4 1239 46 17646232 10.1378/chest.07-0013

[16] Grigoriu BD Scherpereel A Devos P Utility of osteopontin and serum mesothelin in malignant pleural mesothelioma diagnosis and prognosis assessment Clin Cancer Res 2007 13 10 2928 35 17504993 10.1158/1078-0432.CCR-06-2144

[17] Di Serio F Fontana A Loizzi M Mesothelin family proteins and diagnosis of mesothelioma: analytical evaluation of an automated immunoassay and preliminary clinical results Clin Chem Lab Med 2007 45 5 634 8 17484626 10.1515/CCLM.2007.112

[18] Fuhrman C Duche JC Chouaid C Use of tumor markers for differential diagnosis of mesothelioma and secondary pleural malignancies Clin Biochem 2000 33 5 405 10 11018693 10.1016/s0009-9120(00)00157-0

[19] Pass HI Wali A Tang N Soluble mesothelin-related peptide level elevation in mesothelioma serum and pleural effusions Ann Thorac Surg 2008 85 1 265 72discussion 272. 18154821 10.1016/j.athoracsur.2007.07.042

[20] Rodriguez Portal JA Rodriguez Becerra E Rodriguez Rodriguez D Serum levels of soluble mesothelin-related peptides in malignant and nonmalignant asbestos-related pleural disease: relation with past asbestos exposure Cancer Epidemiol Biomarkers Prev 2009 18 2 646 50 19190155 10.1158/1055-9965.EPI-08-0422

[21] Hillerdal G Malignant mesothelioma 1982: review of 4710 published cases Br J Dis Chest 1983 77 4 321 43 6357260

[22] Hollevoet K Bernard D De Geeter F Glomerular filtration rate is a confounder for the measurement of soluble mesothelin in serum Clin Chem 2009 55 7 1431 3 19406915 10.1373/clinchem.2008.121913

[23] Tramonti G Ferdeghini M Donadio C Renal function and serum concentration of five tumor markers (TATI, SCC, CYFRA21-1, TPA, and TPS) in patients without evidence of neoplasia Cancer Detect Prev 2000 24 1 86 90 10757127

[24] Thorpe JD Duan X Forrest R Effects of blood collection conditions on ovarian cancer serum markers PLoS ONE 2007 2 12 e1281 18060075 10.1371/journal.pone.0001281 PMC2093996

[25] Roe OD Creaney J Lundgren S Mesothelin-related predictive and prognostic factors in malignant mesothelioma: a nested case-control study Lung Cancer 2008 61 2 235 43 18281122 10.1016/j.lungcan.2007.12.025

[26] Gao YC Yuan ZB Yang YD Lu HK Effect of freeze-thaw cycles on serum measurements of AFP, CEA, CA125 and CA19-9 Scand J Clin Lab Invest 2007 67 7 741 7 17852813 10.1080/00365510701297480

